# Borderline personality disorder and thyroid diseases: a Mendelian randomization study

**DOI:** 10.3389/fendo.2023.1259520

**Published:** 2023-10-03

**Authors:** Qian Wang, Peijin Li, Shuo Qi, Jiaojiao Yuan, Zhiguo Ding

**Affiliations:** ^1^ Department of Thyropathy, Dongzhimen Hospital, Beijing University of Chinese Medicine, Beijing, China; ^2^ Department of Thyropathy, Sunsimiao Hospital, Beijing University of Chinese Medicine, Tongchuan, Shanxi, China; ^3^ Department of Oncology, Dongzhimen Hospital, Beijing University of Chinese Medicine, Beijing, China

**Keywords:** borderline personality disorder, thyroid disease, Mendelian randomization, causal correlation, GWAS

## Abstract

**Background:**

Previous studies have shown that there is a correlation between diseases of the thyroid gland and mental illnesses; however, any causal relationship between them remains unclear. This study aimed to evaluate the causal relationship between borderline personality disorder and four thyroid diseases.

**Methods:**

The causal relationship was inferred using double-sample Mendelian randomization analysis of appropriate instrumental variables from genome-wide association studies. We calculated the estimated value of the effect using various statistical methods.

**Results:**

Borderline personality disorder was a risk factor for non-toxic single thyroid nodules with each increase in standard deviation increasing the risk of a non-toxic single thyroid nodule by 1.13 times (odds ratio = 1.131; 95% confidence interval, 1.006-1.270; P=0.039). There was no evidence of a correlation between borderline personality disorder and hyperthyroidism/thyrotoxicosis, hypothyroidism, and autoimmune thyroiditis.

**Conclusion:**

This study showed that there is a positive causal correlation between borderline personality disorder and non-toxic single thyroid nodules but not with other thyroid diseases. This means that thyroid status should be monitored in patients with borderline personality disorder. However, the possibility of a causal relationship between other mental illnesses and thyroid diseases requires further research.

## 1 Introduction

Thyroid disease is characterized by abnormal morphology or dysfunction of the thyroid, such as in hyperthyroidism/thyrotoxicosis, hypothyroidism, subacute thyroiditis, autoimmune thyroiditis, thyroid nodules, and thyroid cancer (1–5). Thyroid disease is more common in women, and the impact on the health of women is greater than that of men. A decade ago, the estimated global prevalence of patients with thyroid disease reached approximately 200 million; today, the number is even greater (6). Current studies suggest that the factors associated with thyroid disease include heredity, trace elements (commonly iodine and selenium), vitamins, infection, and radiation (7–9). Some studies have shown that the emergence of thyroid diseases, especially thyroid disorders, can have a negative impact on emotional stability and aggravate existing mental illnesses (10, 11). Notably, our team has found that patients with thyroid disease tend to exhibit one or more negative emotions, such as mania, anxiety, depression, fear, and obsessive-compulsive attitudes and behaviors (unpublished work).

Borderline personality disorder, also known as emotional instability personality disorder, is a common mental disorder mainly characterized by emotion, difficulty with interpersonal relationships and self-image, and behavioral instability accompanied by various impulsive behaviors (12, 13). Notably, although borderline personality disorder is often misdiagnosed as anxiety, depression, and other mental disorders because of the unstable emotional state symptom, it is more serious than these mental disorders. Thus, we hypothesized that there might be a causal relationship between mental disorders and thyroid diseases.

Therefore, this study aimed to assess the potential causal relationship between borderline personality disorder, selected as the exposure, and non-toxic single thyroid nodules, hyperthyroidism/thyrotoxicosis, hypothyroidism and autoimmune thyroiditis, selected as outcomes, to determine whether thyroid status should be monitored in patients with borderline personality disorder.

## 2 Materials and methods

### 2.1 Study design

This study utilized the Mendelian randomization (MR) analysis, which provides a feasible method for our research (14). MR analysis studies the causal relationship between exposure and outcome by selecting appropriate genetic variation as instrumental variables (IVs) (15). Compared with other research methods, the MR can overcome the influence of confounding factors and reverse causality (16, 17).

**Figure 1:**
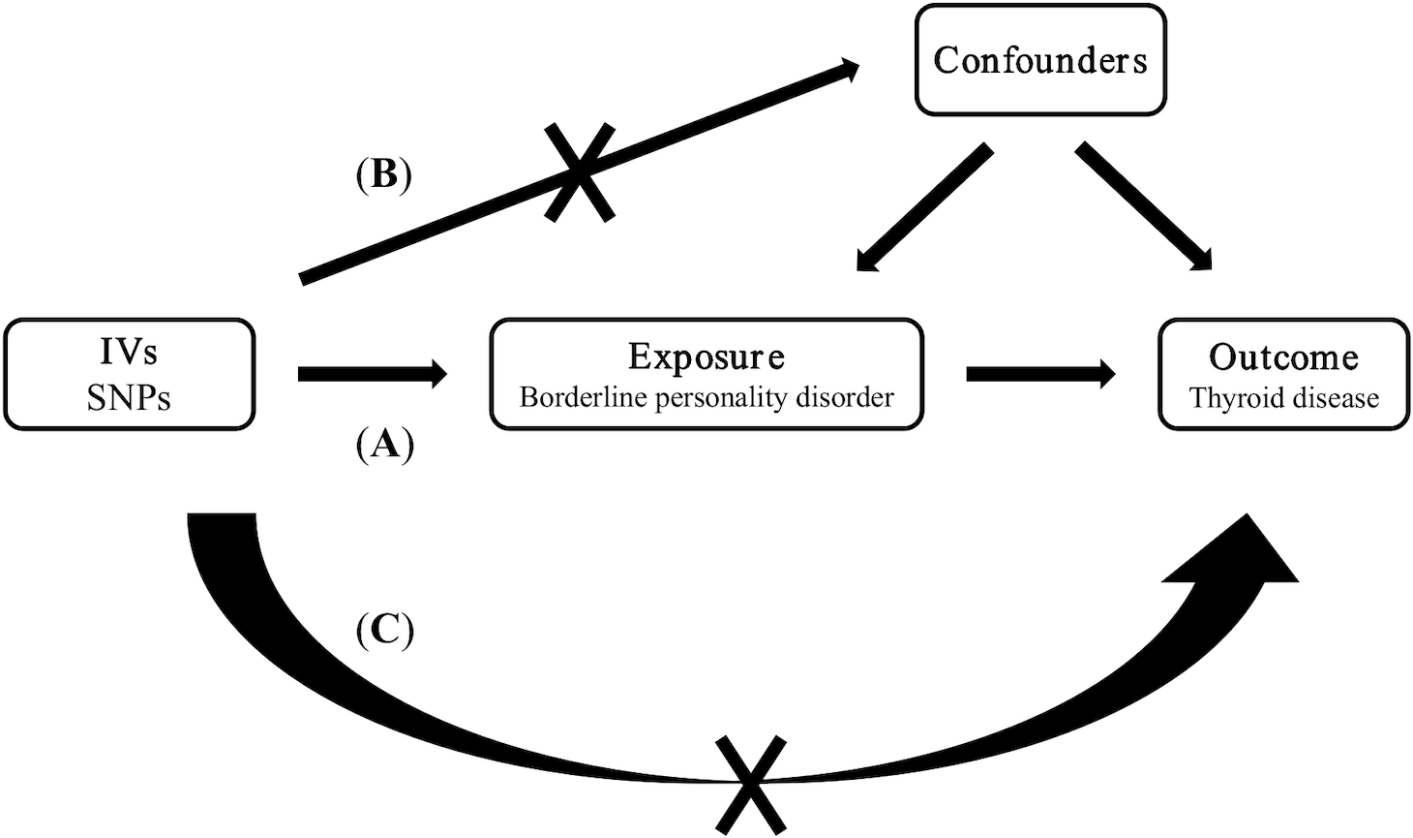
Three key assumptions of Mendelian randomization studies. **(A)** SNP is closely associated with borderline personality disorder; **(B)** SNPs are not associated with confounders; **(C)** SNPs affect thyroid disease only through borderline personality disorder. SNP: single nucleotide polymorphism.

Our study employed a two-sample MR analysis to evaluate the causal relationship between borderline personality disorder and four thyroid diseases. **Figure 1** shows the three key hypotheses in MR analysis as follows (18): (**A**) single nucleotide polymorphism (SNP) is closely associated with borderline personality disorder; (**B**) SNP is independent of all known confounding factors; (**C**) SNP affects thyroid disease only through the presence of borderline personality disorder (**Figure 1**). Our study results were reported in accordance with MR-STROBE guidelines (19).

### 2.2 Ethics statement

All the data used in this study are de-identified public data and have been ethically approved by the appropriate institutions; hence, no additional ethical approval was required.

### 2.3 Data sources

The data in this study were obtained from IEU openGwas project database (https://gwas.mrcieu.ac.uk/). The GWAS ID of borderline personality disorder is finn-b-F5_EMOPER (n=214,816), non-toxic single thyroid nodule is finn-b-E4_GOITRENOD (n=188,805), hyperthyroidism or thyrotoxicosis is ukb-b-20289 (n=462,933), hypothyroidism is ukb-b-4226 (n=463,010), and autoimmune thyroiditis is finn-b-E4_THYROIDITAUTOIM (n=187,928). The populations of the database are from Europe and include both men and women. **Supplementary Material 1** shows the other information in the database.

### 2.4 The choice of SNP

Our study used the following four processes to screen the appropriate SNP. First, we retrieved each SNP in the borderline personality disorder data set in the PhenoScannerV2 database (a database of human genotype-phenotype associations) to eliminate the SNP that may affect the outcome index by affecting other confounding factors. Second, to obtain more data for screening, we regarded P<1E-5 as a significant criterion for screening SNP in borderline personality disorder at the genome-wide level (20). Third, based on the linkage disequilibrium analysis, we speculated that there was a significant linkage disequilibrium of SNP in kb=10000 and R^2^>0.001; hence, we removed it (21). Finally, we tested the strength of each tool variable. When the statistic F was >10, we considered the SNP to be a strong tool variable with a small weak instrument bias (22).

The SNP that met the above screening was used as a standard tool variable for MR analysis. Additionally, we performed effect allele alignment before starting the analysis to remove all SNP with palindromic structure.

### 2.5 Statistical analysis

Statistical analyses were performed with R software version _4.2.3 (R Foundation for Statistical Computing, Vienna, Austria) using the R package (Rpackage “TwoSampleMR”). We considered that there was statistical significance in the causal effect between exposure factors and outcome indicators when P<0.05.

#### 2.5.1 MR analysis

The MR analysis method included inverse variance weighting (IVW), MR-Egger regression, weighted median (WM), simple mode, and weighted mode, in which the IVW was our first choice. However, when the heterogeneity was large, we used WM or IVW under the random effect model, and we used MR-Egger regression as the main analysis method when there was horizontal multiplicity (23, 24). Taking the IVW method as an example, we used the Wald ratio method to correlate a single SNP and then selected a fixed or random effect model to summarize the effects of multiple sites using meta according to the size of its heterogeneity. The odd ratio (OR) and 95% confidence interval (CI) indicated the effect. Simple mode and weighted mode analyses were used as supplements.

#### 2.5.2 Sensitivity analysis

The Q test of Cochrane was used to evaluate the heterogeneity among IVs. We considered that there is a strong heterogeneity among the IVs when P<0.05. The intercept of MR-Egger regression was used to evaluate whether there was horizontal multiplicity among multiple IVs, which was indicated at P<0.05. The SNP with large differences was removed using the MR-Pleiotropy RESidual Sum and Outlier test to further reduce this multiplicity level. Moreover, the horizontal multiplicity after elimination and statistical differences in its existence before and after elimination were detected. The leave-one-out method was also used to determine whether the selected IV was stable and reliable.

## 3 Results

### 3.1 The choice of SNP

All included studies were published between 2018 and 2021 and comprised European participants (**Supplementary Material 1**). Overall, 30 tool variables met the our SNP selection criteria, and all F statistics were >10 (**Supplementary Material 2**).

### 3.2 Results of MR and sensitivity analyses

**Figure 2:**
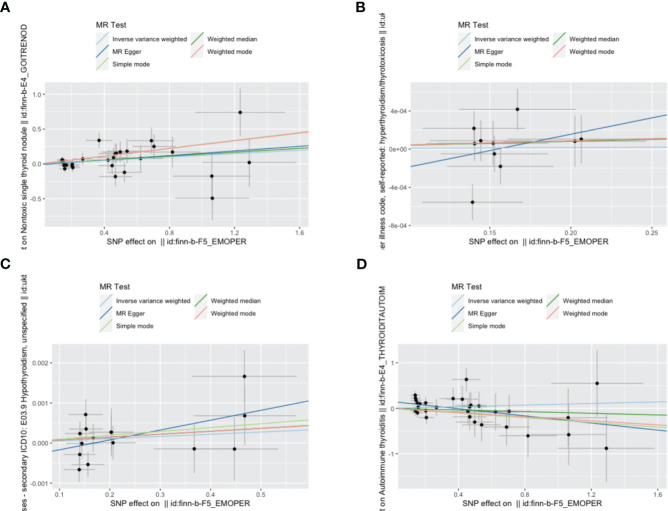
Scatter plot of the association between borderline personality disorder and thyroid disease. **(A)** a single non-toxic thyroid nodule; **(B)** hyperthyroidism/thyrotoxicosis; **(C)** hypothyroidism; **(D)** autoimmune thyroiditis. Each black dot represents a SNP, plotted from the SNP estimate for borderline personality disorder and the SNP estimate for thyroid disease risk, with a standard error bar. The slope of the line corresponds to a causal estimate using each of the different methods. SNP, single nucleotide polymorphism.

**Figure 3:**
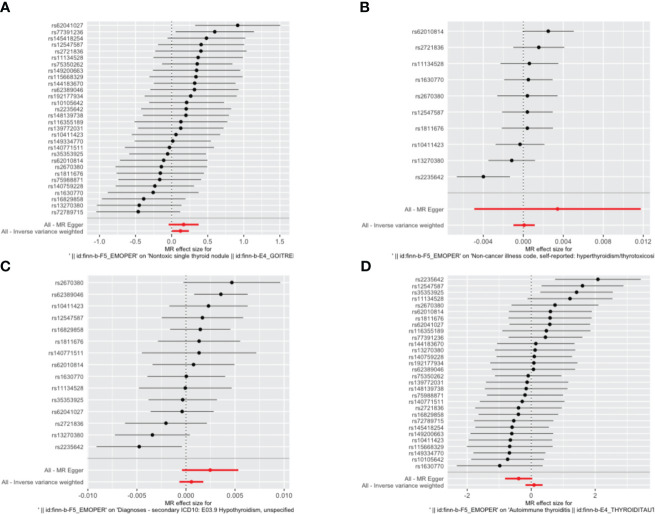
Forest plot of the association between borderline personality disorder and thyroid disease. **(A)** a single non-toxic thyroid nodule; **(B)** hyperthyroidism/thyrotoxicosis; **(C)** hypothyroidism; **(D)** autoimmune thyroiditis. Dots and bars represent causal estimates of the risk of thyroid disease with borderline personality disorder.

**Figure 4:**
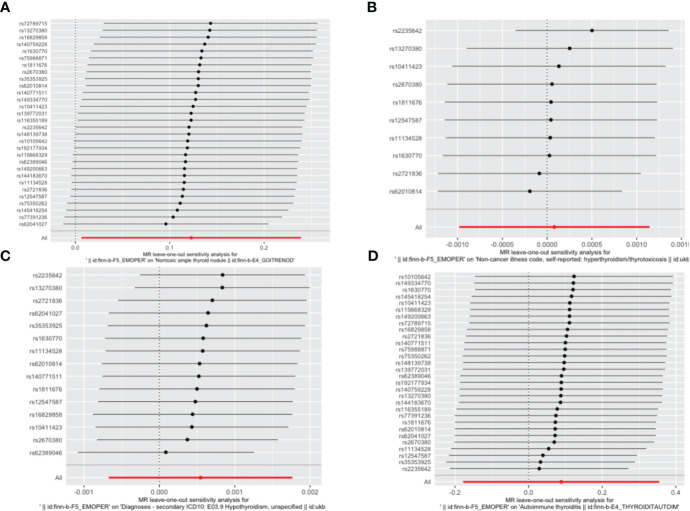
Leave-one-out sensitivity analysis of the association between borderline personality disorder and thyroid disease. **(A)** a single non-toxic thyroid nodule; **(B)** hyperthyroidism/thyrotoxicosis; **(C)** hypothyroidism; **(D)** autoimmune thyroiditis. The dot and bar indicate the estimates and 95% confidence interval when the specific single nucleotide polymorphism is removed.

**Figure 5:**
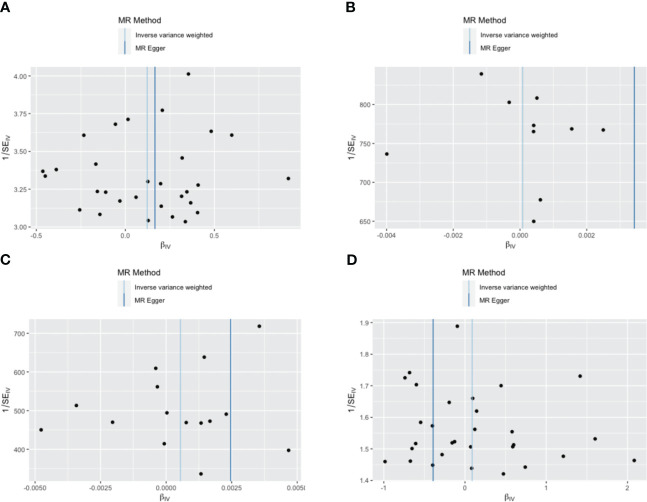
Funnel plot of the association between borderline personality disorder and thyroid disease. **(A)** a single non-toxic thyroid nodule; **(B)** hyperthyroidism/thyrotoxicosis; **(C)** hypothyroidism; **(D)** autoimmune thyroiditis. Each black dot indicates a single nucleotide polymorphism.

IVW, as our primary analysis method, showed that the presence of genetically predicted borderline personality disorder was associated with an increased risk of thyroid nodules (odds ratio (OR)=1.131; 95% confidence interval (CI), 1.006–1.270; P=0.039), and the OR of other MR analysis methods also showed a similar trend (**Supplementary Materials 3**, **4** and **Figures 2**–**5**). No association was observed between borderline personality disorder and hyperthyroidism/thyrotoxicosis, hypothyroidism, and autoimmune thyroiditis using MR analysis (**Supplementary Materials 3**, **4** and **Figures 2**–**5**).

## 4 Discussion

Our study evaluated the causal relationship between borderline personality disorder and four common thyroid diseases, and the results showed that borderline personality disorder has a positive causal relationship with a single non-toxic thyroid nodule, but not with the other three thyroid diseases.

This finding contrasts with our clinical experience, which suggests that patients with hyperthyroidism and hypothyroidism tend to have more diverse psychiatric symptoms than individuals with non-toxic thyroid nodules. Previous studies have shown that individuals with non-toxic single thyroid nodules exhibit different psychiatric symptoms, primarily negative emotions (10, 11). The resultant chronic stress state can lead to changes in neurological, immune, and endocrine functions, consequently leading to changes in thyroid stimulating hormone (TSH) levels. Long-term instability of TSH levels may lead to the formation of thyroid nodules. Hyperthyroidism and hypothyroidism can induce a variety of psychiatric symptoms with differences depending on the disease. Patients with hypothyroidism are more likely to exhibit depression and anxiety, and the “mucinous edematous madness” symptom is extremely rare, while patients with hyperthyroidism are more irritable, nervous, and excited (11, 25–28). Therefore, borderline personality disorder as an exposure factor may not accurately reflect the clinical situation of various thyroid diseases. Additionally, clinical experience is subjective and sometimes not complete.

Some studies have shown that thyroid disease, especially thyroid dysfunction or autoimmune thyroid disease, is associated with negative emotions or mental illnesses; however, the nature of their relationship remains unclear (29, 30). The prevailing hypothesis suggests that thyroid diseases often manifest in varying degrees of mental disorders. Nevertheless, this hypothesis does not answer a crucial question: whether mental disorders occur before or after thyroid disease. Our study demonstrates that borderline personality disorder as a risk factor for non-toxic single thyroid nodules can precede and lead to its emergence.

In this study, we chose borderline personality disorder as the exposure variable because it is more similar to the diverse psychiatric symptoms of patients with thyroid disease and has a lower diagnostic bias than other mental illnesses. We could not include thyroid cancer as an outcome variable as intended because there is no shared SNP in borderline personality disorder and thyroid cancer.

This study has some limitations. First, although the statistics of each tool variable F was >10, it cannot completely overcome the bias caused by weak IVs. Second, although the sensitivity analysis results did not indicate the existence of multiple effects, it is difficult to rule out the potential multiple effects in MR analysis completely. Third, the patients in the GWAS database are only Europeans; hence, extending this conclusion to people of other regions is challenging and perhaps inappropriate. Fourth, the statistical methods in MR analysis are strict and conservative; consequently, the real causal relationship between exposure and outcome might have been ignored.

In conclusion, this study showed that a positive causal correlation exists between borderline personality disorder and non-toxic single thyroid nodules. Therefore, this study suggests that treatment for borderline personality disorder may reduce the risk of developing non-toxic single thyroid nodules and we should consider performing routine monitoring of thyroid status in patients with borderline personality disorder. In addition, considering the similarity of mental illnesses, we speculate that there is a causal relationship between other mental illnesses and thyroid diseases, which we will investigate in future studies.

## Data availability statement

The original contributions presented in the study are included in the article/**Supplementary Material**. Further inquiries can be directed to the corresponding author.

## Ethics statement

Ethical review and approval was not required for the study on human participants in accordance with the local legislation and institutional requirements. Written informed consent for participation was not required for this study in accordance with the national legislation and the institutional requirements.

## Author contributions

QW: Conceptualization, Data curation, Writing – original draft. PL: Formal Analysis, Software, Writing – review & editing. SQ: Writing – review & editing. JY: Visualization, Writing – review & editing. ZD: Funding acquisition, Supervision, Writing – review & editing.
