# Supplementary material for: Borderline personality disorder and thyroid diseases: a Mendelian randomization study
Source: Front Endocrinol (Lausanne). 2023 Oct 3;14:1259520. doi: 10.3389/fendo.2023.1259520 (PMC10579900; doi:10.3389/fendo.2023.1259520)
Supplement: Supplementary file 1 [file Table_1.docx]

Supplementary Material

# Borderline personality disorder and thyroid disease

| Trait | Year | Author | Population | Sample size | n case | n control | Number of SNPs |
| --- | --- | --- | --- | --- | --- | --- | --- |
| Borderline personality disorder | 2021 | NA | European | 214,816 | 2,637 | 212,179 | 16,380,456 |
| Nontoxic single thyroid nodule | 2021 | NA | European | 188,805 | 1,121 | 187,684 | 16,380,359 |
| Hyperthyroidism/Thyrotoxicosis | 2018 | Ben Elsworth | European | 462,933 | 3,545 | 459,388 | 9,851,867 |
| Hypothyroidism | 2018 | Ben Elsworth | European | 463,010 | 9,674 | 453,336 | 9,851,867 |
| Autoimmune thyroiditis | 2021 | NA | European | 187,928 | 244 | 187,684 | 16,380,358 |

# Single nucleotide polymorphisms used as instrumental variables in the Mendelian randomization analyses of borderline personality disorder

| SNP | Chr | EA | NEA | Beta | SE | EAF | F |
| --- | --- | --- | --- | --- | --- | --- | --- |
| rs1630770 | 1 | G | A | -0.2064 | 0.0456 | 0.8814 | 20.48753463 |
| rs192177934 | 1 | C | G | 0.2671 | 0.0605 | 0.06537 | 19.49113039 |
| rs75350262 | 1 | A | G | 0.705 | 0.1319 | 0.01472 | 28.56857925 |
| rs145418254 | 2 | T | C | 0.6892 | 0.1414 | 0.0126 | 23.75700662 |
| rs149200663 | 2 | T | C | 0.4975 | 0.1126 | 0.02072 | 19.52132937 |
| rs16829858 | 2 | G | A | 0.4678 | 0.1012 | 0.02472 | 21.36778031 |
| rs115668329 | 3 | G | T | 0.5393 | 0.1167 | 0.01652 | 21.35596146 |
| rs139772031 | 3 | G | T | 0.6228 | 0.1366 | 0.01399 | 20.78719112 |
| rs140759228 | 4 | A | G | 0.5234 | 0.1066 | 0.02326 | 24.10754728 |
| rs116355189 | 5 | A | G | 0.4229 | 0.0954 | 0.02605 | 19.65072694 |
| rs11134528 | 5 | T | C | 0.1444 | 0.0314 | 0.3566 | 21.14828188 |
| rs72789715 | 5 | A | T | 1.0635 | 0.2286 | 0.005206 | 21.64325301 |
| rs148139738 | 5 | T | C | -0.4565 | 0.102 | 0.02463 | 20.0300125 |
| rs2670380 | 6 | G | A | -0.152 | 0.0342 | 0.7486 | 19.75308642 |
| rs62389046 | 6 | T | G | 0.4673 | 0.1041 | 0.02253 | 20.15069841 |
| rs12547587 | 8 | G | A | 0.1406 | 0.0297 | 0.4736 | 22.41081976 |
| rs13270380 | 8 | A | G | -0.1565 | 0.0327 | 0.7033 | 22.90515202 |
| rs10105642 | 8 | A | G | 0.8186 | 0.1728 | 0.009151 | 22.44171302 |
| rs77391236 | 9 | T | C | 1.2347 | 0.2764 | 0.003564 | 19.95477425 |
| rs1811676 | 10 | G | A | 0.2025 | 0.044 | 0.1354 | 21.18091426 |
| rs144183670 | 10 | A | G | 0.4746 | 0.0936 | 0.02578 | 25.71010026 |
| rs140771511 | 11 | C | T | 0.203 | 0.0438 | 0.1322 | 21.48047372 |
| rs35353925 | 11 | A | T | 0.4475 | 0.0868 | 0.03246 | 26.57948911 |
| rs75988871 | 12 | C | T | 1.0603 | 0.239 | 0.00465 | 19.68165981 |
| rs149334770 | 15 | T | G | 1.2903 | 0.2905 | 0.003983 | 19.72827536 |
| rs62041027 | 15 | T | C | -0.3676 | 0.0809 | 0.03775 | 20.64685759 |
| rs62010814 | 15 | G | A | 0.167 | 0.0361 | 0.2224 | 21.4002348 |
| rs2235642 | 16 | C | T | -0.1393 | 0.031 | 0.3614 | 20.19197711 |
| rs2721836 | 17 | G | T | -0.1402 | 0.0317 | 0.6654 | 19.56038969 |
| rs10411423 | 19 | G | A | -0.1528 | 0.0332 | 0.7244 | 21.18217448 |

# MR results between borderline personality disorder and thyroid disease

| Outcomes | No. of SNPs | Method | OR(95%CI) | *p* |
| --- | --- | --- | --- | --- |
| Nontoxic single thyroid nodule | 30 | Inverse variance weighted | 1.131 (1.006-1.270) | 0.039 |
|  |  | MR Egger | 1.181 (0.960-1.453) | 0.128 |
|  |  | Weighted median | 1.150 (0.984-1.344) | 0.080 |
|  |  | Simple mode | 1.324 (0.944-1.858) | 0.114 |
|  |  | Weighted mode | 1.319 (0.955-1.821) | 0.104 |
| Hyperthyroidism/Thyrotoxicosis | 10 | Inverse variance weighted | 1.000 (0.999-1.001) | 0.882 |
|  |  | MR Egger | 1.003 (0.995-1.012) | 0.443 |
|  |  | Weighted median | 1.000 (0.999-1.002) | 0.475 |
|  |  | Simple mode | 1.000 (0.999-1.002) | 0.625 |
|  |  | Weighted mode | 1.000 (0.999-1.002) | 0.617 |
| Hypothyroidism | 15 | Inverse variance weighted | 1.001 (0.999-1.002) | 0.379 |
|  |  | MR Egger | 1.002 (1.000-1.005) | 0.117 |
|  |  | Weighted median | 1.001 (0.999-1.002) | 0.318 |
|  |  | Simple mode | 1.001 (0.998-1.003) | 0.463 |
|  |  | Weighted mode | 1.001 (0.998-1.003) | 0.547 |
| Autoimmune thyroiditis | 30 | Inverse variance weighted | 1.093 (0.837-1.426) | 0.515 |
|  |  | MR Egger | 0.675 (0.442-1.032) | 0.080 |
|  |  | Weighted median | 0.913 (0.659-1.266) | 0.586 |
|  |  | Simple mode | 0.772 (0.382-1.561) | 0.478 |
|  |  | Weighted mode | 0.796 (0.416-1.522) | 0.496 |

# Sensitivity analysis between borderline personality disorder and thyroid disease

| Outcomes | Method | Heterogeneity | | Pleiotropy | | MR-PRESSO |
| --- | --- | --- | --- | --- | --- | --- |
|  |  | Q | *p* | Intercept | *p* |  |
| Nontoxic single thyroid nodule | MR Egger | 34.05 | 0.20 | -1.34E-02 | 0.62 | 0.218 |
|  | Inverse variance weighted | 34.35 | 0.23 |  |  |  |
| Hyperthyroidism/Thyrotoxicosis | MR Egger | 14.19 | 0.08 | -5.29E-04 | 0.45 | 0.089 |
|  | Inverse variance weighted | 15.31 | 0.08 |  |  |  |
| Hypothyroidism | MR Egger | 18.13 | 0.15 | -4.15E-04 | 0.18 | 0.093 |
|  | Inverse variance weighted | 20.99 | 0.10 |  |  |  |
| Autoimmune thyroiditis | MR Egger | 31.31 | 0.30 | 1.49E-01 | 0.01 | 0.108 |
|  | Inverse variance weighted | 39.47 | 0.09 |  |  |  |
